# Vaccination coverage and breakthrough infections of COVID-19 during the second wave among staff of selected medical institutions in India

**DOI:** 10.1371/journal.pgph.0000946

**Published:** 2023-04-07

**Authors:** Manju Rahi, Chander Prakash Yadav, Sundus Shafat Ahmad, Nitika, Payal Das, Shweta Sharma, Rajendra Kumar Baharia, Debdutta Bhattacharya, Pradeep Deshmukh, Amey Dhatrak, Sandeep Dogra, Alex Eapen, Pawan Goel, Nafis Faizi, Siraj A. Khan, Sanjay Kumar Kochar, Aditya Kochar, Ashwani Kumar, Anuj Mundra, Rahul Narang, Kanwar Narain, Krishna Pandey, Sanghamitra Pati, Pankaja Raghav, Ritesh Ranjha, Salman Shah, Kuldeep Singh, Piyoosh Kumar Singh, Raj Kumar Singh, Vijesh Shreedhar Kuttiatt, Ravinder Soni, Uragayala Sreehari, Sumit Malhotra, Amit Sharma

**Affiliations:** 1 Indian Council of Medical Research, New Delhi, India; 2 Academy of Scientific and Innovative Research (AcSIR), Ghaziabad, Uttar Pradesh, India; 3 ICMR- National Institute of Cancer Prevention & Research, Noida, Uttar Pradesh, India; 4 New Delhi and its Field Units (FUs), ICMR- National Institute of Malaria Research, New Delhi, India; 5 Regional Medical Research Centre, Bhubaneshwar, Odisha, India; 6 All India Institute of Medical Sciences, Nagpur, Maharshtra, India; 7 Mahatma Gandhi Institute of Medical Sciences, Wardha, Maharashtra, India; 8 Government Medical College, Jammu, Jammu and Kashmir, India; 9 Shaheed Hasan Khan Mewati Government Medical College, Mewat, Haryana, India; 10 Aligarh Muslim University, Aligarh, Uttar Pradesh, India; 11 Regional Medical Research Centre, Dibrugarh, Assam, India; 12 Sardar Patel Medical College, Bikaner, Rajasthan, India; 13 Vector Control Research Centre, Puducherry, India; 14 All India Institute of Medical Sciences, Bibinagar, Telangana, India; 15 Rajendra Memorial Research Institute of Medical Sciences, Patna, Bihar, India; 16 All India Institute of Medical Sciences, Jodhpur, Rajasthan, India; 17 Dayanand Medical College, Ludhiana, Punjab, India; 18 All India Institute of Medical Sciences, New Delhi, India; 19 International Centre for Genetic Engineering and Biotechnology, New Delhi, India; New York University Grossman School of Medicine, UNITED STATES

## Abstract

India experienced the second wave of SARS-CoV-2 infection from April 3 to June 10, 2021. During the second wave, Delta variant B.1617.2 emerged as the predominant strain, spiking cases from 12.5 million to 29.3 million (cumulative) by the end of the surge in India. Vaccines against COVID-19 are a potent tool to control and end the pandemic in addition to other control measures. India rolled out its vaccination programme on January 16, 2021, initially with two vaccines that were given emergency authorization–Covaxin (BBV152) and Covishield (ChAdOx1 nCoV- 19). Vaccination was initially started for the elderly (60+) and front-line workers and then gradually opened to different age groups. The second wave hit when vaccination was picking up pace in India. There were instances of vaccinated people (fully and partially) getting infected, and reinfections were also reported. We undertook a survey of staff (front line health care workers and supporting) of 15 medical colleges and research institutes across India to assess the vaccination coverage, incidence of breakthrough infections, and reinfections among them from June 2 to July 10, 2021. A total of 1876 staff participated, and 1484 forms were selected for analysis after removing duplicates and erroneous entries (n = 392). We found that among the respondents at the time of response, 17.6% were unvaccinated, 19.8% were partially vaccinated (received the first dose), and 62.5% were fully vaccinated (received both doses). Incidence of breakthrough infections was 8.7% among the 801 individuals (70/801) tested at least 14 days after the 2nd dose of vaccine. Eight participants reported reinfection in the overall infected group and reinfection incidence rate was 5.1%. Out of (N = 349) infected individuals 243 (69.6%) were unvaccinated and 106 (30.3%) were vaccinated. Our findings reveal the protective effect of vaccination and its role as an essential tool in the struggle against this pandemic.

## Introduction

A large section of the population worldwide suffered because of the COVID-19 pandemic in terms of significant morbidity and mortality. During the second wave, India witnessed a sudden spurt of cases and deaths due to COVID-19 from April 3 2021 to June 10 2021. This wave was different from the first wave in terms of infectivity rates, age groups affected, percentage of severe infections, rate of hospitalizations and mortality [1, 2]. Worldwide the health care systems were under the pressure of COVID-19 cases as hospitals suffered from shortages of beds, Intensive Care Unit (ICU) space, oxygen supply and health care personnel [3]. As of April 3 2021, India reported 12.5 million positive cases (cumulative) and 0.1 million deaths due to COVID-19. By June 10, 2021, the number of positive cases and deaths reported rose to 29.3 million and 0.3 million respectively [4].

Apart from appropriate COVID-19 Non-Pharmacological Interventions (NPI), which includes maintaining social distance, using masks, appropriate ventilation and hand hygiene, vaccines were a potent tool in the campaign against COVID-19. The government of India gave emergency use authorization to Covaxin (BBV152) (manufactured by Bharat Biotech) and Covishield (ChAdOx1 nCoV- 19) (manufactured by Serum Institute of India) with efficacy of ~77% and ~70% respectively [5, 6]. The vaccination programme was launched on January 16, 2021, and the roll-out was in a phased manner in a sequence of prioritization of the elderly and front-line workers. Subsequently, vaccination was opened up to the rest of the age groups [7]. “Vaccination coverage” is the proportion of the eligible populations who have received a specific vaccine [8]. As of January 1, 2023, ~2201 million COVID-19 vaccine doses (1027million first doses, 951 million second doses and 222 million booster doses) have been administered and over 90% of the Indian population has successfully received 2 doses of vaccine [9]. It was anticipated that the introduction of vaccination will slow down the pace of the pandemic and significantly reduce the rate of hospitalizations, as the vaccines have been shown to reduce the severity of the disease, if not the transmission of infection [10]. Hence, as the vaccines were not 100% effective, cases were reported among fully vaccinated individuals. Furthermore, reinfection among previously infected cases were also reported, suggesting waning natural immunity or immune escape by mutant strains of the virus. Breakthrough infection, defined as infection occurring after two weeks (14 days) of the second vaccine dose against SARS-CoV-2, was also a cause of concern [8, 11]. The Indian Council of Medical Research (ICMR), the nodal research body of the Government of India, reported the rate of infections after the COVID-19 vaccine as less than 0.05% [12]. Vaishya et al reported that in one New Delhi hospital ~76% of HCWs who were infected had breakthrough infections [13]. Mukherjee et al 2021 defined reinfection as a laboratory confirmed SARS-CoV-2 infection at an interval of at least 102 days with one negative molecular test in between after being negative for the first infection. The study further reported a rate of 4.5% in HCWs and the general population [14]. The above-mentioned studies helped set an epidemiological definition of SARS-Cov-2 reinfections from India. The present study was undertaken to assess the vaccination coverage (either of the two available COVID-19 vaccines in India at that time), the occurrence of infections among the fully vaccinated (i.e., breakthrough infections), and reinfection incidence among the staff of selected medical colleges and research institutes across India.

## Methods

### Study design and data collection

**Fig 1:**
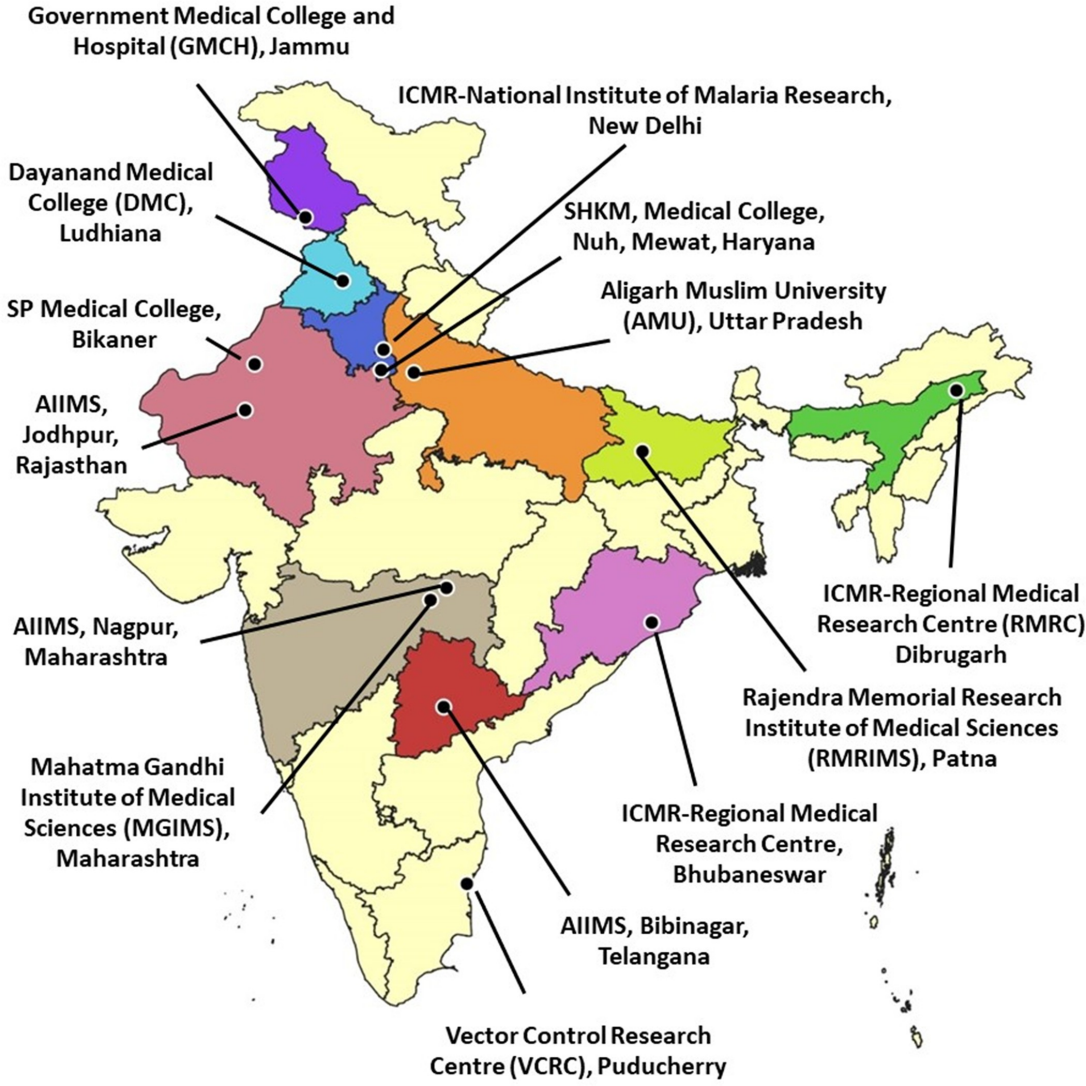
Map showing the participating institutes in the cross-sectional, online population-based survey for healthcare workers.

This cross-sectional online study collected information from healthcare workers and supporting staff from June 2 to July 10, 2021. The data were collected through an online questionnaire from 15 participating medical teaching and research institutes. The institutes involved are located in different parts of the country and are shown in Fig 1. The access link (available at: https://forms.gle/yYQB3FGywwxbcdt16) was provided to the partner institutes through mail, text, and WhatsApp messages. The institutes were encouraged to widely disseminate the information about the study and also the questionnaire to their staff involved directly in patient care and their other staff members. The participants visited the URL on any device such as mobile phones, desktop computers, or laptops to respond to the questionnaire. On clicking on the link, they were informed about the study objectives, data confidentiality, and consent form. The respondents were requested to participate in the survey by completing the questionnaire without any time restrictions. The inclusion criteria for participants were: (1) 18 years of age or older; (2) being able to read, comprehend, self-administer and respond to the questionnaire. The participation in the survey was voluntary.

The questionnaire consisted of four parts: Section A was on demographic information, Section B on COVID-19 infection and re-infection, Section C on vaccination, and Section D on information on breakthrough infection. The questions posed were close-ended to minimize discrepancies. In section A, the study participants were required to fill in the information regarding name, age, gender, blood group, name of the institute, designation and contact details. The next part of the form comprised of questions on COVID-19 infection in the past (from the onset of pandemic till reporting by the respondent), and other necessary details like date, symptoms, method of diagnosis, hospitalization status, number of days hospitalized, comorbidities, and results from NAATs (Nucleic Acid Amplification Tests) on recovery. Then, the information was sought on the re-infection: date, severity, symptoms, results of NAATs and hospitalization status. Also, the information on vaccination with either Covaxin or Covishield was sought with the date of inoculation. Thereafter, information on COVID-19 infections occurring after receipt of COVID-19 vaccine was collected with the details of dates and symptomatology. Laboratory confirmed SARS-CoV-2 infection detected by molecular tests like RT-PCR /TruNat/CBNAAT were included as true infections.

### Statistical analysis

The data was collected using Google Forms and exported to MS Excel and analyzed in Stata 15.0. Before formal statistical analysis, the data were scrutinized and cleaned using logical check. Discrepancies and erroneous entries were resolved by contacting participants via phone calls. Those who did not respond by phone were excluded from the analysis. Descriptive statistics in terms of n (%) was used for expressing the qualitative data and mean plus-minus standard deviation was used for expressing quantitative data. Logistic regression was performed to assess the factors associated with the uptake of COVID-19 vaccination, and magnitude of association was reported using odds ratio (OR) and a 95% confidence interval.

### Ethics

ICMR- National Institute of Malaria Research has obtained approval from the Institutional Ethics committee (IEC) with relevant documents after institute’s scientific advisory committee gave approval. Ethics approval has been obtained from ethics committees of all participating institutes. All respondents provided an e-consent before submitting their responses. E-consent form is provided in S1 File.

## Results

**Fig 2:**
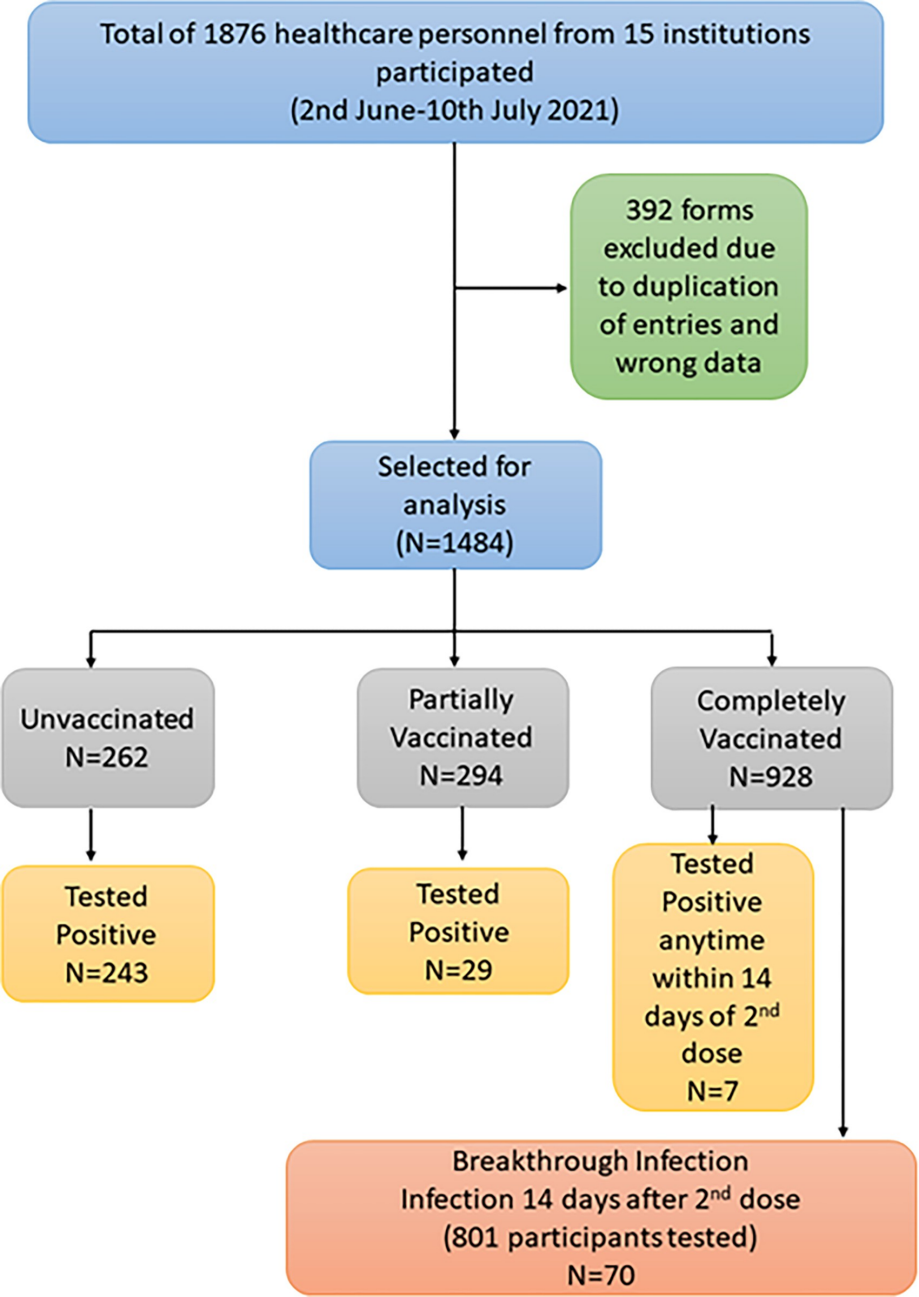
Study flowchart.

A total of 1876 healthcare personnel filled out the online questionnaire from 15 participating institutes out of which 1484 were included in this analysis and 392 were excluded owing to wrong entries or errors in data that could not be rectified (see flow chart in Fig 2). Table 1 shows the distribution and profile of respondents included in the study. Results given in Table 1 show that out of the 1484 respondents 1035 (69.8%) were from the ten government medical colleges and hospitals and remaining 449 (30.2%) were from the five ICMR institutes. Medical healthcare workers (doctors, nurses, paramedics) were 609 (41%), non-medical (research faculty, students and staff) were 317 (21.4%) and clerical/administrative/supportive staff were 314 (21.2%). The mean age standard deviation (SD) in respondents’ years was 35.1 (±11.3) years. The majority of respondents 1170 (78.8%) belonged to age band of 18–45 years, followed by 46–60 years in which there were 314 (21.2%) respondents. More than half of the participants were females, i.e., 817 out of 1484 (55.1%). The maximum number of respondents (1414; 95.3%) did not report any co-morbidities while 70 (4.8%) reported having two or more comorbidities. Almost one-fourth (349/1484) of the participants experienced COVID-19 infection at some point of time (from the onset of pandemic till the time of reporting by the respondent) and only eight (0.5%) participants were infected twice during the period, indicating a reinfection rate of 5.1%. Also, 1135 (76.3%) remained uninfected till the reporting time (July 10, 2021; 00:00 hours). Among the COVID-19 infected respondents, hospitalization was reported by only 52 (14.9%) and the rest (297; 85.1%) recovered at their homes. Among the participants, 62.5% (928/1484) received both doses (complete vaccination) of the available vaccines, 294 (19.8%) received only one dose of the vaccine, while 262 (17.6%) were unvaccinated at the time of reporting. In our study, among the 1222 participants who received either one or two doses of the approved vaccines, we found that 85.5% (1046 of 1222) received Covishield and 14.4% (176 of 1222) received Covaxin.

**Table 1:** Distribution and characteristics of participants (N = 1484).

Characteristics	N (%)
**Institute**	
MGIMS, Sevagram, Wardha	455 (30.7)
DMC, Ludhiana	321 (21.6)
ICMR-VCRC	164 (11.1)
ICMR-RMRC, Dibrugarh	114 (7.7)
ICMR-NIMR, New Delhi	104 (7.0)
Kasturba hospital, Sewagram	83 (5.6)
ICMR-RMRC, Bhubaneshwar	59 (4.0)
AIIMS, Nagpur	57 (3.8)
AMU Medical College, Aligarh	57 (3.8)
AIIMS, Hyderabad	32 (2.2)
Others	38 (2.6)
**Designation**	
Nursing Staff	286 (19.3)
Supporting Staff	245 (16.5)
Medical students	188 (12.7)
Non-medical students	177 (11.9)
Research staff	140 (9.4)
Medical Faculty	103 (6.9)
Clerical/Administrative staff	69 (4.7)
Laboratory staff	32 (2.2)
Did not disclose their designation	244 (16.4)
**Age, years (Mean ±SD)**	**35.1±11.3**
18–45	1170 (78.8)
46–60	314 (21.2)
**Sex**	
Female	817 (55.1)
Male	667 (44.9)
**Comorbidities**	
`No	1414 (95.3)
Yes	70 (4.8)
**Blood group**	
A	318 (21.4)
AB	153 (10.3)
B	482 (32.5)
O	470 (31.7)
Don’t know	61 (4.1)
**COVID-19 Infection**	
No infection	1135 (76.5)
Single infection	340 (22.9)
Re-infection	8 (0.5)
**Hospitalization due to COVID (N = 349)**	
No	297 (85.1)
Yes	52 (14.9)
**Vaccination status**	
Unvaccinated	262 (17.6)
Partially Vaccinated	294 (19.8)
Completely Vaccinated	928 (62.5)
**Vaccine Type among vaccinated (N = 1223)**	
Covaxin	177 (14.5)
Covishield	1046 (85.5)

**Fig 3:**
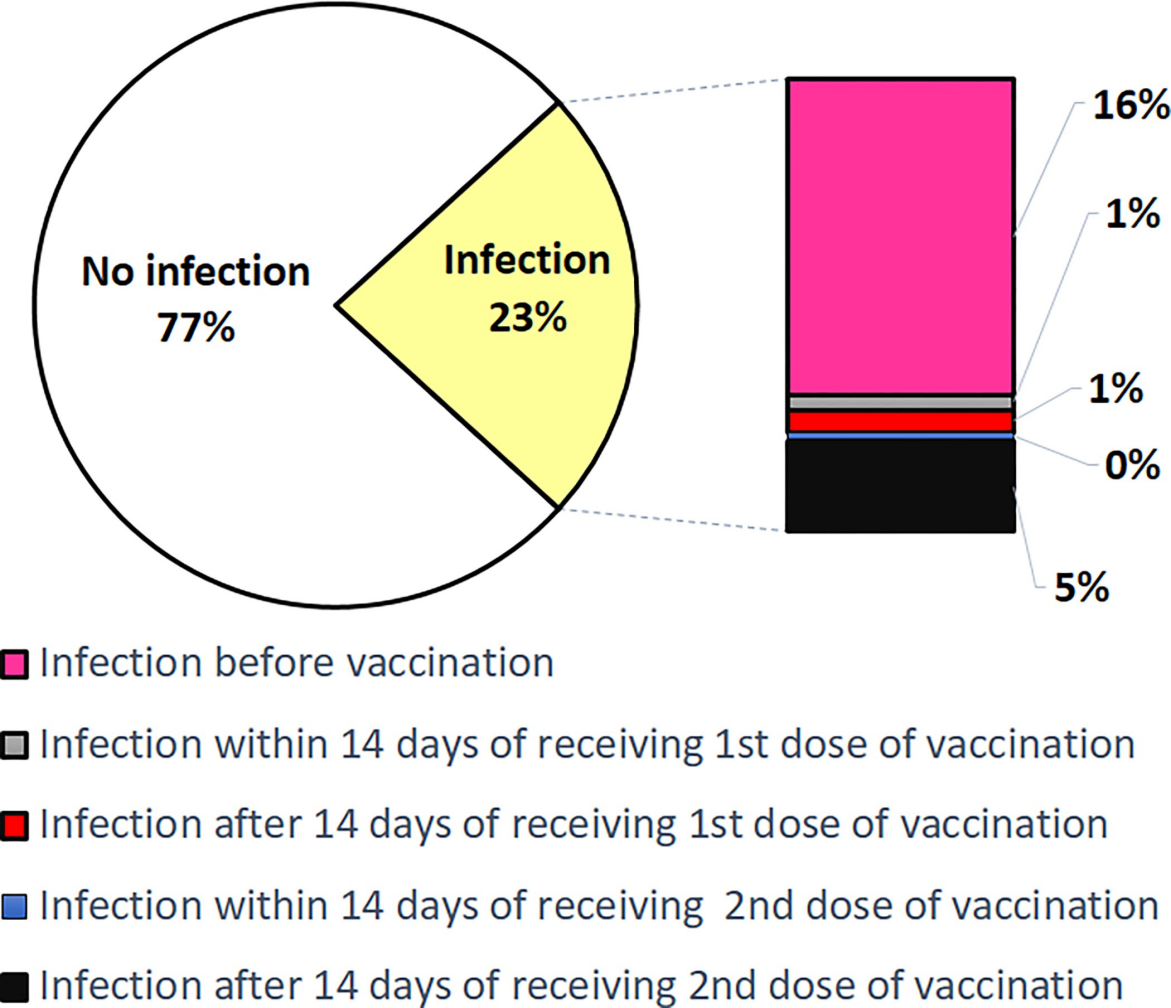
Infection distribution based on vaccination.

Further analysis was done with regard to participant characteristics of COVID-19 infected individuals (349) in the context of vaccination (Table 2). Among the 349 infected participants, the maximum fell in the age band of 18–45 years (279; 79.9%), followed by 46–60 years (70; 20.1%). The majority of non-infected respondents belonged to the age band of 18–45 years (891; 78.5%), followed by 46–60 years (244; 21.5%). While no comorbidities were reported in the non-infected participants, 70 of 349 (20.1%) infected participants reported comorbidities. Of the total 349 infected individuals, 255 (73.06%) were infected before active vaccination and 24 (6.8%) were infected after partial immunization. Additionally, 70 of the 349 (4.7%) became SARS-CoV-2 positive after a span of atleast 14 days from the day of the second dose. Further details of results are given in Table 2 and in S1 Table. Fig 3 depicts the distribution of the participants according to the COVID-19 infection.

**Table 2:** Participant characteristics for COVID-19 infections in context of vaccination (N = 1484).

Characteristics	No infection	Infection before vaccination	Infection after partial vaccination	Infection after vaccination	Total Infection
n (%)	1135(76.5)	255(17.2)	24(1.6)	70(4.7)	349 (23.5)
[95 CI]	[74–78]	[15–19]	[0.8–2.2]	[3.6–5.8]	[21–26]
Age, years					
Mean ± SD	34.9±11.5	35.7±10.5	35.4±12.3	35.4±12.3	35.8±10.5
18–45	891 (78.5)	203 (79.6)	19 (76.2)	57 (81.4)	279(79.9)
46–60	244 (21.5)	52 (20.4)	5 (20.8)	13 (18.6)	70 (20.1)
Gender					
Female	627 (55.2)	144 (56.5)	13 (54.2)	33 (47.1)	190(54.4)
Male	508 (44.8)	111 (43.5)	11 (45.8)	37 (52.9)	159(45.6)
Presence of co-morbidity					
No	1135	201 (78.8)	22 (91.7)	56 (80.0)	279(79.9)
Yes	0	54 (21.2)	2 (8.3)	14 (20.0)	70 (20.1)
Symptomatic status					
Asymptomatic	1135 (100)	43 (16.9)	6 (25.0)	13 (18.6)	62 (17.8)
Symptomatic	0	212 (83.1)	18 (75.0)	57 (81.4)	287(82.2)
Hospitalization due to COVID					
No	1135 (100)	210 (82.3)	21 (87.5)	66 (94.3)	297(85.1)
Yes	0	45 (17.7)	3 (12.5)	4 (5.7)	52 (14.9)
Blood group					
A	251 (22.1)	48 (18.8)	6 (25.0)	13 (18.6)	67 (19.2)
AB	112 (9.9)	31 (12.2)	3 (12.5)	7 (10.0)	41 (11.7)
B	362 (31.9)	86 (33.7)	6 (25.0)	28 (40.0)	120 (34.4)
O	361 (31.8)	83 (32.5)	5 (20.8)	21 (30.0)	109 (31.2)
Don’t know	49 (4.3)	7 (2.7)	4 (16.7)	1 (1.4)	12 (3.4)

Distribution of age category (p-value 0.564), gender (p-value 0.792), and blood group (p-value 0.141) are comparable in first four groups. Distribution of comorbidity, hospitalization and symptomatic status were not assessed as none in the ‘No-infection’ had any comorbidity or symptoms or were admitted to hospital.

Our analysis (Table 2) also produced the incidence of breakthrough infections. Breakthrough infections were based on either or both symptoms and laboratory confirmation. Out of 801 respondents, 70 were infected two weeks post complete vaccination with SARS-CoV 2. This makes the incidence of breakthrough infection 8.7%. Among the participants with breakthrough infection, the majority fell into the age band of 18–45 years (81.4%), followed by 46–60 years (18.6%). The breakthrough infection was slightly more in males (37/70; 52.9%) than in females (33/70; 47.9%). Among the 70 breakthrough infections, 14 (20.0%) respondents reported comorbidities and, 81.4% (57) were symptomatic, but only 4 (5.7%) required hospitalization. Of those who were completely vaccinated, only 6 individuals (2 within 14 days of 2^nd^ dose and 4 after 14 days of 2^nd^ dose) required hospitalization. Out of the 70 breakthrough infections detected in our study, 5 participants were such who reported infection prior to vaccination.

Additional information on the clinical symptoms of the infected cohort is given in S2 Fig. Of the 349 respondents who suffered from COVID-19, 287 (82.2%) were symptomatic. Fever, cough, body ache, sore throat, and headache were the most common but symptoms such as diarrhoea (11; 12.9%), breathlessness (12; 14.1%) and persistent pain or pressure in the chest (9; 10.6%) were reported more frequently in those who got infected prior to vaccination. (S2 Fig).

S2 Table describes the factors associated with the profile of respondents concerning COVID-19 vaccination. Out of 1170, 947 (80.9%) respondents belonging to the 18–45 age group were vaccinated as compared to 275 (87.6%) out of 314 respondents belonging to the age group 46–60. That means the older age group was vaccinated 1.6 times (or odds) more as compared to the younger age group. Gender and comorbidity status did not show any association with vaccination rate. The odds of obtaining vaccination were only 0.33 in those infected before vaccination compared to those who were not infected before vaccination. A complete participant recruitment flowchart is given in S3 Fig.

## Discussion

In our study, coverage of COVID-19 vaccination (Covaxin or Covishield) among healthcare staff, reinfections and post-vaccination occurrence of infections (i.e., breakthrough infections) were evaluated through an online survey. In a study in Delhi hospital in 2021 the authors reported that 280 (85.8%) healthcare staff had taken two doses, while 46 (14.2%) had taken only one dose [16]. Covishield was the predominant vaccine (85.5%) as it was more widely available. As per national data, by January 17, 2021, 60.6% of the eligible Indian population had been vaccinated with one dose and 39.4% had received two doses. These findings corroborate with our results. A large proportion of the population (88.8%) has been vaccinated with COVIDshield, followed by Covaxin and a very small proportion by Sputnik V (Gam-COVID-Vac) [9].

Vaccination was high in the 18–45 year bracket as the people in this age group are the predominant workforce. This is following national trends, according to which around 60% of the vaccinated masses belong to the same age bracket [9]. Although vaccination was initiated first for individuals 60+ in age, India has a substantial proportion of people (~ 42%) belonging to the age bracket of 18–44 years and 36.7% were below 18 years, and 21% of population were above 45 years [15]. Therefore, maximum participation and vaccination were observed among those aged 18–45 years, in absolute numbers. However, if we look at proportions, 80.9% of the 18–45 age group were vaccinated as compared to 87.6% of the 46–60 years age group, owing to higher comorbidities and earlier vaccination in this age group.

Amongst our study population, 55% were females and 45% were males, with a similar distribution in those having received vaccination (Table 2); one or two doses (54.2% females vs 45.8% males). This is in contrast to the proportion in the general population in India, where more males than females have been vaccinated, i.e., as of July 30, 2022, 99,29,63,816 males as compared to females [9]. In a study by Sharma et al (2021), conducted in a teaching hospital in Delhi, India, out of 326 healthcare workers (medical doctors, interns, frontline health workers, lab technicians, and nurses) who were enrolled, 212 (65%) were males and 114 (35%) were females [16]. In another study by Tyagi et al (2021), COVID related data was collected from healthcare workers like doctors, paramedical and other staff from January 16 to May 31, 2021 [17]. The results revealed that 75 (60.9%) were males and 48 (39.0%) were females out of 123 participants. The increased proportion of vaccinated females is increased participation from nurses (19.3%) compared to other staff who were primarily administrative and clerical staff. Other studies had higher proportions of non-nursing staff as compared to ours.

Overall, among the COVID-19 infected participants (Table 2), hospitalization was reported by only 14.9% while the other 85.1% recovered in home-based settings. The study by Vaishya et al (2021) also reported a minor hospitalization rate of 0.06% i.e., only 2 out of 3235 vaccinated HCWs [13]. This is similar to the study conducted by Malhotra et al (2021) which reported only one participant with severe illness out of 1917 [18]. Another study by Malhotra et al (2021) reported the most common symptoms to be fever and cough, which were seen in 70.5% and 52% participants, respectively [19]. This is similar to our findings as fever and cough were our most common symptoms too, followed by body ache, sore throat and headache.

The Government of India adopted a policy that recommended hospital admission only in moderate/ severe COVID-19 infections, i.e., when patients showed danger signs such as unrelenting fever, difficulty in breathing, breathlessness, or a drop in oxygen saturation below 90% [7]. This policy might be the reason why our study and similar studies reported low hospitalization rates. Up to ~80% of the infected population showed symptoms because individuals tend to get tested only when symptomatic. Also, as per policy, only symptomatic contacts were recommended to be tested. Our study also showed how vaccination has a proven protective effect, as vaccinated individuals showed even more reduced hospitalization rates. A study by Kale et al (2021) conducted at Institute of Liver and Billiary Sciences, Delhi reported that unvaccinated individuals were more susceptible to get infected than vaccinated individuals with one or two doses [20]. Not a single vaccinated subject needed Intensive Care Unit (ICU) admission owing to severe disease, and no mortality was reported. On the other hand, there were three severe cases and one death in the unvaccinated group. A study by Schimpff (2021) in Los Angeles, USA reported a relatively small proportion (<10%) of COVID-19 positive individuals who were vaccinated were also hospitalized [21].

In a study in the United Kingdom (UK) conducted between December 2020 and July 2021, 1,240,009 individuals reported a first vaccine dose, of whom 6030 (0·5%) subsequently tested positive for SARS-CoV-2, and 971,504 were reported with second dose, of whom 2370 (0·2%) subsequently tested positive for SARS-CoV-2 [22]. In another study among ~ 100,000 people in the UK during the Delta wave, i.e., June-July 2021, it was observed that fully vaccinated individuals were at a substantially lower (two-thirds less) risk of harbouring the SARS-CoV-2 virus than unvaccinated people [23]. A study by Bergwerket et al, in Israel reported 39 breakthrough cases among 1497 fully vaccinated HCWs [24]. A study by Alishaq et al in Qatar, reported 164 breakthrough infections among 22,247 fully vaccinated HCWs. [25].

Our study reported 70 (8.7%) post-vaccination breakthrough infections, out of which 81.4% (57) were reported from the 18–45 years age group (Table 2). Other Indian researchers have also reported breakthrough infections in fully vaccinated individuals. The study by Tyagi et al, (2021) conducted in a health care facility located in Delhi observed that breakthrough infections were mild in their clinical manifestation [17]. Out of their 123 employees, only 15 (13.3%) experienced breakthrough infections and almost all (14) were mild except one who suffered a serious illness and needed hospitalization. Another cross-sectional study performed by Sharma et al (2021) at a teaching hospital in Delhi reported that ~14% (approximately one in seven healthcare staff) had a breakthrough infection [16]. Gupta et al (2021) reported that out of 677 breakthrough cases from across the country, 53 showed severe symptoms and were hospitalized [12]. Our study highlights the protective effect of vaccination. Out of 349 infected individuals, 243 (69.6%) were unvaccinated. Out of 262 unvaccinated people, 243 (92.7%) were infected. On the other hand, out of 801 individuals, atleast 14 days post 2 doses of vaccines, 70 (8.7%) were infected. Our study reports a reinfection rate of 5.1% which is comparable to the study by Malhotra et al, that reported a reinfection rate of 6.05% [18]. Although we did not conduct any immunological tests ie; antibody titre detection, from among the the 70 breakthrough infections, 5 participants reported infection prior to vaccination; suggesting perhaps a fortified immune response.

Muniz-Diaz et al found that O blood group had a protective effect against COVID-19 [26], and in our study, we found blood group B in a slightly higher preponderance in the infected group as compared to O. In the uninfected population there was no difference. We can attribute our findings to the fact that blood group B was in overall majority in our study population. However, similar studies by Rana et al and Acik and Bankir have reported A, B, and Rh+ are found to be more susceptible to COVID-19 infection, whereas blood groups O, AB, and Rh− are at a lower risk of COVID-19 infection [27, 28].

## Limitations

Our study has few limitations. We might have missed asymptomatic or mild cases and underestimated breakthrough infections as these cases were likely untested. Additionally, all our participants were from hospitals or research centres who presumably had a higher risk of exposure. Also, the survey might have missed individuals who did not have access or the knowledge to use digital tools such as WhatsApp and thus did not participate in this survey. All the questions in our survey were mandatory, and only those forms that were completed and information was cross checked to be correct were used for further analysis. This has lead to us dropping out the participants entries where information was incomplete. Our study was conducted during the summer months hence the impact of seasonality and waning immunity is also considered a limitation. There were certain limitations in this study’s data collection tools too such as not considering positive rapid antigen tests as the diagnostic criteria for COVID-19 infections. In this study, the severity of infection was judged based on hospitalization and symptomatology given that moderate to severe infection cases would have been hospitalized. However, we understand that with this approach we may have missed a fraction of moderate and severe cases that were managed by home isolation because of the non-availability of beds or patient preference.

## Conclusion

Several factors will affect the occurrence of COVID-19 cases in a geographical area. These factors may include the effectiveness of vaccines over time, human behaviour, infection prevention policies, mutated strains and the proportion of immunocompromised people. The B.1.1.529 Omicron variant was classified by WHO as a variant of concern. The variant had been reported in ~77 countries and spread fast. It is understood that this strain (may have) caused less severe COVID-19 symptoms and affected the upper lung regions compared to early variants of the virus [10]. COVID-19 has effects beyond just the symptoms it causes. It has affected economies and led to financial security in people. Fear and panic are associated with the disease [29] and the battle not over. Countries will continue to wage war against COVID-19 pandemic till it is reduced to an endemic infection. Minimizing vaccine hesitancy and universal uptake of vaccines is of paramount importance as vaccination has been acknowledged as the best way to end the pandemic.

Our study has shown how vaccines impart a protective effect and also reduce severity of infection, leading to reduced morbidity, mortality and hospitalizations. Our analysis had certain limitations like potentially missing asymptomatic or mild cases. Also, participants being from hospitals or research institutes had greater access to healthcare facilities than general public, and digital data collection tool limited the participation in the survey.

Breakthrough infections pose a challenge not only for public health but also for healthcare personnel as they are front-line workers. Breakthrough infections should be given special attention for healthcare personnel who manage COVID-19 patients. Well-planned epidemiological studies and genomic surveillance are needed to delineate breakthrough infection risk factors and predictors. Therefore, studies like our current work will be imperative for understanding the trend of vaccination coverage and post-vaccination infection especially in context of the emergence of new variants of concern.

## Supporting information

S1 Fig Distribution of COVID19 infectivity based on blood groups. (TIF)

S2 Fig Symptoms of COVID-19 patients (N = 287). (TIF)

S3 Fig Participant recruitment process. (TIF)

S1 Table Participant characteristics for COVID-19 infections (Breakdown of Table 2 data). (DOCX)

S2 Table Factors associated with COVID-19 vaccination. (DOCX)

S3 Table Infection distribution based on participant designation. (DOCX)

S4 Table Vaccination distribution based on participant designation. (DOCX)

S1 File E-consent form. (PDF)
