# Supplementary figures and images for: Vaccination coverage and breakthrough infections of COVID-19 during the second wave among staff of selected medical institutions in India
Source: PLOS Glob Public Health. 2023 Apr 7;3(4):e0000946. doi: 10.1371/journal.pgph.0000946 (PMC10081792; doi:10.1371/journal.pgph.0000946)

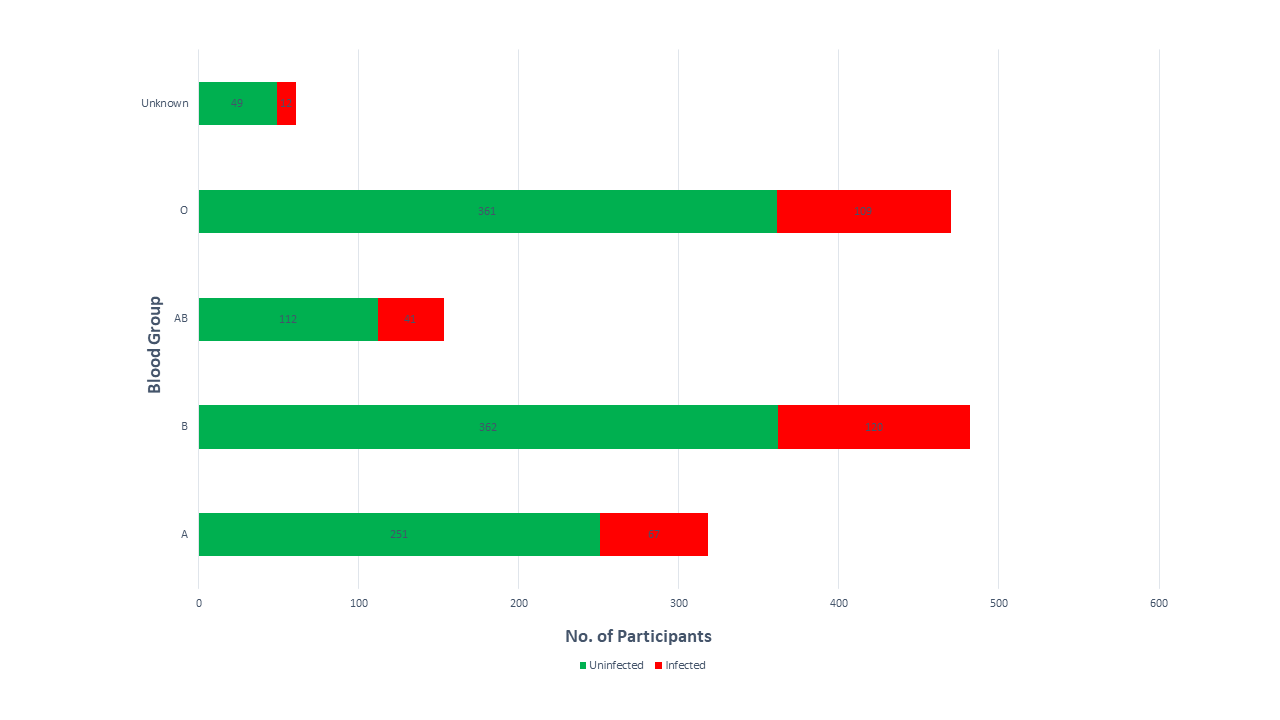

Supplement: S1 Fig — (TIF) [file pgph.0000946.s001.TIF]

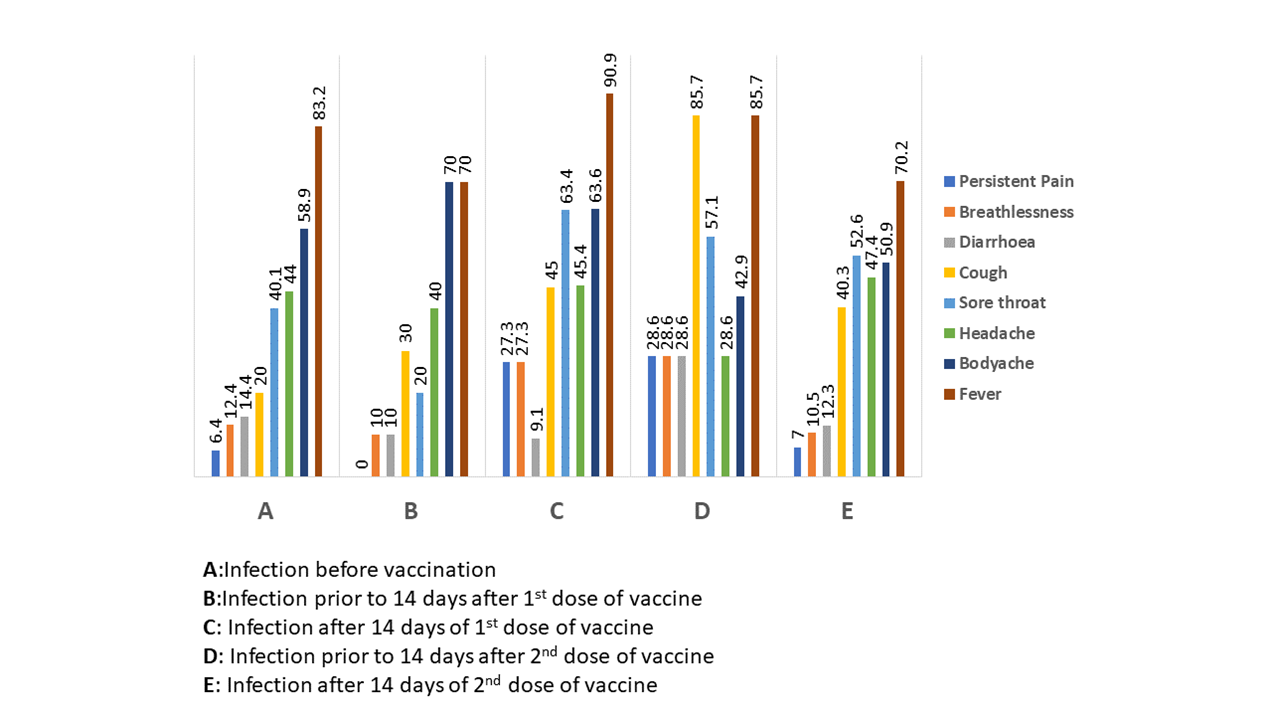

Supplement: S2 Fig — (TIF) [file pgph.0000946.s002.TIF]

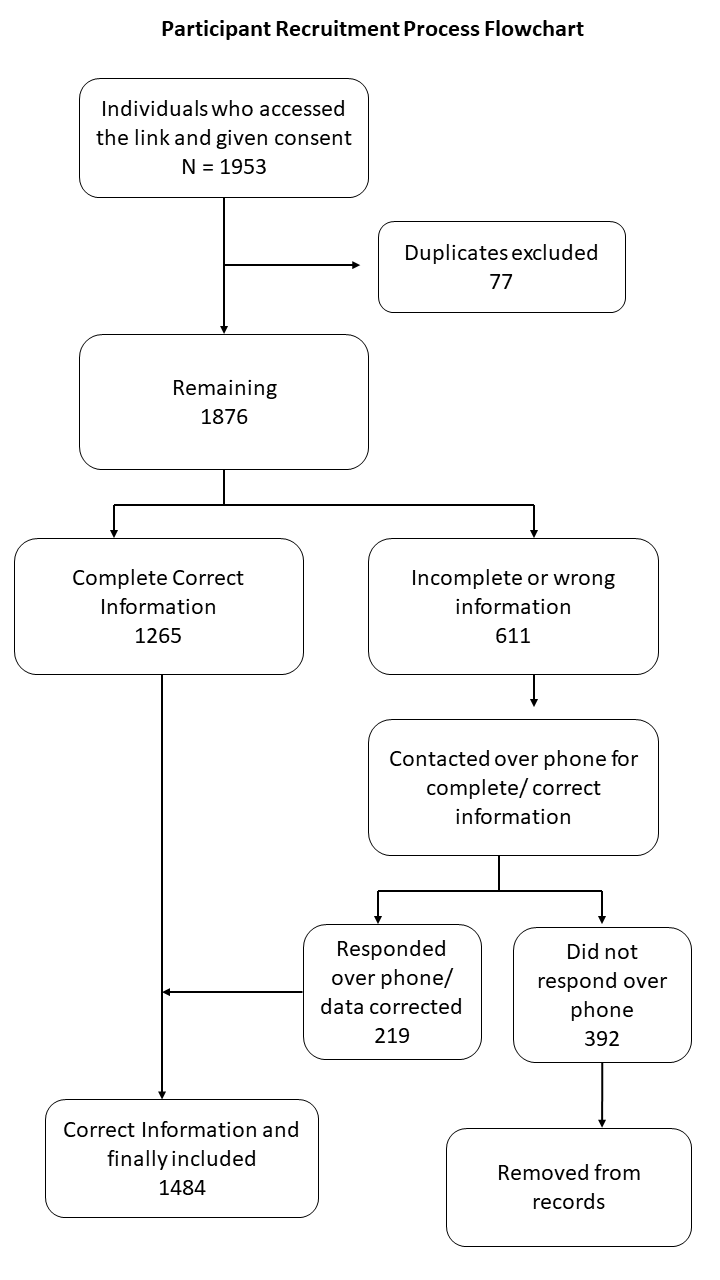

Supplement: S3 Fig — (TIF) [file pgph.0000946.s003.TIF]
