# Supplementary material for: Vaccination coverage and breakthrough infections of COVID-19 during the second wave among staff of selected medical institutions in India
Source: PLOS Glob Public Health. 2023 Apr 7;3(4):e0000946. doi: 10.1371/journal.pgph.0000946 (PMC10081792; doi:10.1371/journal.pgph.0000946)
Supplement: S1 Table — (DOCX) [file pgph.0000946.s004.docx]

**S1 Table – Participant characteristics for COVID-19 infections (breakdown of Table 2 data)**

|  | **Infection before vaccination** | | **Infection after partial vaccination** | |
| --- | --- | --- | --- | --- |
|  | **Infection before vaccination** | **Infection within 14 days of receiving1^st^ dose of vaccination** | **Infection after 14 days of receiving 1^st^ dose of vaccination** | **Infection within 14 days of receiving 2^nd^ dose of vaccination** |
| n (%)  [95 CI] | 243(16.4)  [14-18] | 12(0.8)  [0.4-1.4] | 17(1.1)  [0.7-1.8] | 7(0.5)  [0.2-1.0] |
| Age, years  Mean ± SD  18-45  46-60 | 35.6±10.5  194 (79.8)  49 (20.2) | 38±10.8  9 (75.0)  3 (25.0) | 35.2±13.2  13 (76.5)  4 (23.5) | 35.8±10.7  6 (85.7)  1 (14.3) |
| Gender  Female  Male | 137 (56.4)  106 (43.6) | 7 (58.3)  5 (41.7) | 9 (52.9)  8 (47.1) | 4 (57.1)  3 (42.9) |
| Presence of co-morbidity  No  Yes | 190 (78.2)  53 (21.8) | 11 (91.7)  1 (8.3) | 15 (88.2)  2 (11.8) | 7 (100)  0 |
| Symptomatic status  Asymptomatic  Symptomatic | 41 (16.9)  202 (83.1) | 2 (16.7)  10 (83.3) | 6 (35.3)  11 (64.7) | 0  7 (100) |
| Hospitalization due to covid  No  Yes | 200 (82.3)  43 (17.7) | 10 (83.3)  2 (16.7) | 16 (94.1)  1 (5.9) | 5 (71.4)  2 (28.6) |
| Blood group  A  AB  B  O  Don’t know | 46 (18.9)  29 (11.9)  85 (35.0)  77 (31.7)  6 (2.5) | 2 (16.7)  2 (16.7)  1 (8.3)  6 (50.0)  1 (8.3) | 3 (17.6)  1 (5.9)  4 (23.5)  5 (29.4)  4 (23.5) | 3 (42.8)  2 (28.6)  2 (28.6)  0  0 |
