# Supplementary material for: Vaccination coverage and breakthrough infections of COVID-19 during the second wave among staff of selected medical institutions in India
Source: PLOS Glob Public Health. 2023 Apr 7;3(4):e0000946. doi: 10.1371/journal.pgph.0000946 (PMC10081792; doi:10.1371/journal.pgph.0000946)
Supplement: S2 Table — (DOCX) [file pgph.0000946.s005.docx]

**S2 Table– Factors associated with COVID-19 vaccination**

|  | **N** | **Unvaccinated**  **(N=262)** | **Any dose of vaccination**  **(N=1222)** | **OR**  **(95% CI)** | **P-value** |
| --- | --- | --- | --- | --- | --- |
| **Age, years**  18-45  46-60 | **1170**  **314** | 223 (19.6)  39 (12.4) | 947 (80.9)  275 (87.6) | Ref  1.66 (1.15 to 2.39) | 0.007 |
| **Gender**  Female  Male | **817**  **560** | 155 (19.0)  107 16.0) | 662 (81.0)  560 (84.0) | Ref  1.22 (0.94 to 1.60) | 0.141 |
| **Comorbidities**  No  Yes | **1414**  **70** | 248 (17.5)  14 (20.0) | 1166 (82.5)  56 (80.0) | Ref  0.85 (0.46 to 1.55) | 0.598 |
| **Covid-19 prior to**  **vaccination**  No  Yes | **1241**  **243** | 180 (14.5)  82 (33.7) | 1061(85.5)*  161 (66.3)# | Ref  0.33 (0.24 to 0.45) | <0.001 |

Data are expressed in n (row percentages)

*Were not infected before receiving vaccination (one dose or both doses)

# Were infected before receiving vaccination (one dose or both doses)
