# Supplementary material for: Vaccination coverage and breakthrough infections of COVID-19 during the second wave among staff of selected medical institutions in India
Source: PLOS Glob Public Health. 2023 Apr 7;3(4):e0000946. doi: 10.1371/journal.pgph.0000946 (PMC10081792; doi:10.1371/journal.pgph.0000946)
Supplement: S3 Table — (DOCX) [file pgph.0000946.s006.docx]

**S3 Table: Infection distribution based on participant designation**

| **Designation** | **No infection** | **Infection before vaccination** | **Infection after partial vaccination** | **Infection after vaccination** |
| --- | --- | --- | --- | --- |
| Clerical/Administrative (N=69) | 54 (78.3) | 13 (18.8) | 1 (1.4) | 1 (1.4) |
| Medical faculty (N=103) | 71 (68.9) | 19 (18.4) | 1 (1.0) | 12 (11.6) |
| Laboratory staff (N=32) | 23 (71.9) | 6 (18.7) | 0 | 3 (9. 4) |
| Medical students (N=188) | 146 (77.7) | 30 (16.0) | 3 (1.6) | 9 (4.8) |
| Nursing staff (N=286) | 196 (68.5) | 71 (24.8) | 7 (2.4) | 12 (4.2) |
| Research staff (N=140) | 104 (74.3) | 34 (24.3) | 1 (0.7) | 1 (0.7) |
| Non-medical students (N=177) | 158 (89.3) | 15 (8.5) | 1 (0.6) | 3 (1.7) |
| Supporting Staff (N=245) | 203 (82.7) | 34 (13.9) | 2 (0.8) | 6 (2.4) |
|  |  |  |  |  |
