# Supplementary material for: Vaccination coverage and breakthrough infections of COVID-19 during the second wave among staff of selected medical institutions in India
Source: PLOS Glob Public Health. 2023 Apr 7;3(4):e0000946. doi: 10.1371/journal.pgph.0000946 (PMC10081792; doi:10.1371/journal.pgph.0000946)
Supplement: S4 Table — (DOCX) [file pgph.0000946.s007.docx]

**S4 Table: Vaccination distribution based on participant designation**

| **Designation** | **Not Vaccinated** | **Partial vaccinated** | **Fully vaccinated** |
| --- | --- | --- | --- |
| Clerical/Administrative (n=69) | 4 (5.8%) | 24 (34.78%) | 41 (59.42%) |
| Faculty (n=103) | 5 (4.85%) | 8 (7.77%) | 90 (87.38) |
| Laboratory Staff (n=32) | 1 (3.13%) | 9 (28.12%) | 22 (68.75%) |
| Medical Social Worker (n=9) | 0 | 0 | 9 (100%) |
| Medical Student (n=188) | 22 (11.7%) | 34 (18.09%) | 132 (70.21%) |
| Nursing Staff (n=286) | 74 (25.87%) | 49 (17.13%) | 163 (56.99%) |
| Research Staff (n=37) | 12 (32.43%) | 10 (27.03%) | 15 (40.54%) |
| Scientists (n=34) | 3 (8.82%) | 9 (26.47%) | 22 (64.71%) |
| Non-medical Students (n=172) | 28 (16.28%) | 23 (13.37%) | 121 (70.35%) |
| Supporting Staff (n=236) | 44 (18.64%) | 66 (27.97%) | 126 (53.39%) |
| Technical Staff (n=69) | 3 (4.35%) | 25 (36.23%) | 41 (59.42%) |
| Technical Trainees (n=5) | 0 | 0 | 5 (100%) |

- Percentages are calculated row wise
