# Supplementary material for: Vaccination coverage and breakthrough infections of COVID-19 during the second wave among staff of selected medical institutions in India
Source: PLOS Glob Public Health. 2023 Apr 7;3(4):e0000946. doi: 10.1371/journal.pgph.0000946 (PMC10081792; doi:10.1371/journal.pgph.0000946)
Supplement: S1 File — (PDF) [file pgph.0000946.s008.pdf]

Questions Responses 2,040 Settings

आई सी एम आर–राष्ट्रीय मलेरिया अनुसंधान संस्थान
स्वास्थ्य अनुसंधान विभाग, स्वास्थ्य एवं
परिवार कल्याण मंत्रालय, भारत सरकार

ICMR–National Institute of Malaria Research
Department of Health Research, Ministry of Health & Family Welfare, Government of India

विश्व स्वास्थ्य संगठन जनस्वास्थ्य कीटनाशकों के प्रयोगशाला परीक्षण एवं मूल्यांकन हेतु सहयोग केंद्र
WHO Collaborating Centre for Laboratory Testing and Evaluation of Public Health Pesticides

Section 1 of 8

Information on Covid-19 vaccination, breakthrough infections and reinfections

Informed Consent

You are invited to participate in a web-based online survey on "Assessment of vaccination coverage and incidence of breakthrough infections and reinfections among staff of select medical institutions". This is a research project being conducted by ICMR-National Institute of Malaria Research, New Delhi. It should take approximately 10 minutes to complete the survey.

BACKGROUND AND OBJECTIVE
It has been more than a year since the SARS-CoV-2 pandemic wreaked havoc in India. Currently, India is in the midst of a second wave which is more deadly than the first one. Vaccine is said to be protective against severe disease and mortality and also reduce the transmission of COVID-19. Healthcare staff (working in COVID-19) in medical colleges and research institutes were prioritized as front line and health care workers and offered the vaccine since the drive began. In India, vaccination is voluntary and not mandatory; people were informed and motivated for getting the vaccine as soon as possible.
In this regard, we propose to carry out a voluntary online survey of healthcare staff with the aim to assess the COVID-19 vaccination coverage among the respondents of the participating institutes and estimate the incidence of COVID-19 infection and reinfection amongst the vaccinated which will help to study the association between the asymptomatic and symptomatic and severity of COVID-19 among the above two groups.

PARTICIPATION
Your participation in this survey is voluntary. You may refuse to take part in the research or exit the survey at any time. You are free to decline to answer any particular question you do not wish to answer for any reason.

CONFIDENTIALITY
Your survey answers will be automatically sent to a Google excel sheet where data will be stored in a password protected. All your responses will remain anonymous. No names or identifying information would be included in any publications or presentations based on these data, and your responses to this survey will remain confidential.

IF YOU HAVE ANY QUESTIONS ABOUT THIS STUDY, PLEASE CONTACT
Email address: director@mrcindia.org

ELECTRONIC CONSENT (Please select your choice below. Clicking on the "Agree" button indicates that 1. You have read the above information 2. You voluntarily agree to participate)

Drop-down

1. Agree — Go to section 2 (General information)
2. Disagree — Submit form
3. Add option

Required
